# De Novo Reconstruction of 3D Human Facial Images from DNA Sequence

**DOI:** 10.1002/advs.202414507

**Published:** 2025-05-07

**Authors:** Mingqi Jiao, Jiarui Li, Bingxu Zhong, Siyuan Du, Shuning Li, Manfei Zhang, Qibin Zhang, Zhongming Liang, Fan Liu, Chunman Zuo, Sijia Wang, Luonan Chen

**Affiliations:** ^1^ Key Laboratory of Systems Health Science of Zhejiang Province Hangzhou Institute for Advanced Study University of Chinese Academy of Sciences Chinese Academy of Sciences Hangzhou 310024 China; ^2^ CAS Key Laboratory of Computational Biology Shanghai Institute of Nutrition and Health University of Chinese Academy of Sciences Chinese Academy of Sciences Shanghai 200031 China; ^3^ Bio‐X Institutes Key Laboratory for the Genetics of Developmental and Neuropsychiatric Disorders Ministry of Education Shanghai Jiao Tong University Shanghai 200030 China; ^4^ Naif Arab University for Security Sciences Riyadh 11433 Saudi Arabia; ^5^ School of Life Sciences Sun Yat‐sen University Guangzhou 510275 China; ^6^ Taizhou Institute of Health Sciences Fudan University Taizhou Jiangsu 200433 China; ^7^ Center for Excellence in Animal Evolution and Genetics Chinese Academy of Sciences Kunming 650223 China; ^8^ Key Laboratory of Systems Biology Shanghai Institute of Biochemistry and Cell Biology Center for Excellence in Molecular Cell Science Chinese Academy of Sciences Shanghai 200031 China; ^9^ School of Mathematical Sciences and School of AI Shanghai Jiao Tong University Shanghai 200240 China

**Keywords:** artificial Intelligence in genetics, deep multimodal integration, DNA‐guided 3D face generation, genetic phenotyping, representational learning

## Abstract

Facial morphology is a distinctive biometric marker, offering invaluable insights into personal identity, especially in forensic science. In the context of high‐throughput sequencing, the reconstruction of 3D human facial images from DNA is becoming a revolutionary approach for identifying individuals based on unknown biological specimens. Inspired by artificial intelligence techniques in text‐to‐image synthesis, it proposes Difface, a multi‐modality model designed to reconstruct 3D facial images only from DNA. Specifically, Difface first utilizes a transformer and a spiral convolution network to map high‐dimensional Single Nucleotide Polymorphisms and 3D facial images to the same low‐dimensional features, respectively, while establishing the association between both modalities in the latent features in a contrastive manner; and then incorporates a diffusion model to reconstruct facial structures from the characteristics of SNPs. Applying Difface to the Han Chinese database with 9,674 paired SNP phenotypes and 3D facial images demonstrates excellent performance in DNA‐to‐3D image alignment and reconstruction and characterizes the individual genomics. Also, including phenotype information in Difface further improves the quality of 3D reconstruction, i.e. Difface can generate 3D facial images of individuals solely from their DNA data, projecting their appearance at various future ages. This work represents pioneer research in de novo generating human facial images from individual genomics information.

## Graphical abstract

This study introduces Difface, a novel deep‐learning model for reconstructing 3D facial images only from DNA data. By integrating transformer networks, spiral convolutions, and a diffusion model, Difface directly aligns high‐dimensional SNP data with 3D facial structures. It achieves a superior accuracy in *de novo* facial feature reconstruction and DNA‐to‐3D image alignment, showcasing its potential in forensic science and personalized medicine.

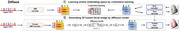

## 1 Introduction

Facial morphology represents a unique and genetically inherited biometric characteristic, providing critical insights for personal identification in forensic applications. DNA profiling, a fundamental strategy in forensic science, which identifies individuals by comparing unknown biological samples (profiled DNA) with known biological specimens,^[^ ^1^, ^2^, ^3^ ^]^ playing a critical role in identification. It is the gold standard in forensic investigations, providing definitive results where traditional methods may falter. As a complementary method, DNA phenotyping analyzes physical traits such as hair and eye color, aiding targeted forensic investigations and streamlining the process. A frontier lies in extracting facial imagery from genetic sequences, poised to transform identification strategies. However, the human face, composed of eyes, nose, chin, and mouth, is shaped by both genetic and environmental factors. Especially in genetically homogeneous Chinese populations, the shape and pigmentation of the face are less variable than in immigrant populations such as those in the United States. Therefore, reconstructing facial images only from genetic data are challenging^4^ ^]^: 1) limited knowledge of facial genetics; 2) technological difficulties due to high‐dimensional data and small sample size. Currently, many studies have been developed to study facial genetics.^[^ ^5^, ^6^, ^7^, ^8^, ^9^, ^10^, ^11^, ^12^, ^13^, ^14^, ^15^, ^16^ ^]^ Huang et al. introduce a genome‐wide association study of facial morphology that identifies novel genetic loci in Han Chinese.^[^ ^17^ ^]^ Xiong et al. introduce C‐GWAS, refining genome‐wide association studies to identify new genetic loci for facial traits.^[^ ^18^ ^]^ Zhang et al.’s research^[^ ^19^ ^]^ conducts genome‐wide analysis to explore facial morphology variations between East Asian and European populations, enhancing our understanding of genetic influences across ancestries. Overall, our understanding of facial genetics remains incomplete. On the other hand, some studies have tried to construct the association between genetics data and facial phenotypes. Specifically, Gurovich et al.^[^ ^20^ ^]^ present a framework for facial image analysis by identifying genetic disorders. Claes et al.^[^ ^21^ ^]^ uncover relations between facial variation and the effects of sex, genomic ancestry using bootstrapped response‐based imputation modeling (BRIM). Sero et al.^[^ ^22^ ^]^ and Mahdi et al.^[^ ^23^ ^]^ proposed models to match facial images with unidentified demographic and genomic attributes of identified individuals. Facial image classification is also an active area of research in machine learning (i.e., 3D face based prediction of sex,^[^ ^24^ ^]^ age,^[^ ^25^ ^]^ ancestry,^[^ ^26^ ^]^ and sexual orientation^[^ ^27^ ^]^). Although these methods have revealed interesting findings, they cannot generate facial images from genetic data.

Inspired by the advanced techniques in text‐to‐image alignment and synthesis,^[^ ^28^, ^29^, ^30^ ^]^ we proposed Difface which explores both generative diffusion process and contrastive multi‐modal alignment, to enable direct to de novo 3D facial image reconstruction from SNPs. Applying Difface to our Han Chinese database with the paired SNP phenotypes and 3D facial images of 9674 individuals demonstrates excellent performance in DNA‐to‐3D image alignment and reconstruction and characterizes the individual of genomics. Further augmenting the model's performance, integrating auxiliary phenotype data—encompassing age, gender, and Body Mass Index (BMI) into Difface significantly enhanced the precision of the 3D facial reconstructions. In addition, we also identified new SNPs features significantly associated with the related facial regions. This multifaceted work not only validates the robustness of Difface but also underscores its potential as a versatile tool in personalized medicine and forensic identification.

However, this groundbreaking technology challenges current conceptions of genomic privacy, raising significant ethical and legal implications. By associating de‐identified genomic data with phenotypic measurements, our research aims to facilitate a deeper discussion on the impacts of DNA phenotyping. We invite commentary and deliberation on the implications of these findings for genomics research, investigatory practices, and broader societal ethical considerations. Although some scholars have addressed the implications of DNA phenotyping, this work emphasizes the necessity of thorough analysis to navigate the complex interplay of innovation and privacy, ultimately contributing to more informed and ethical genomic research.

## 2 Results

### 2.1 Overview of Difface

**Figure 1:**
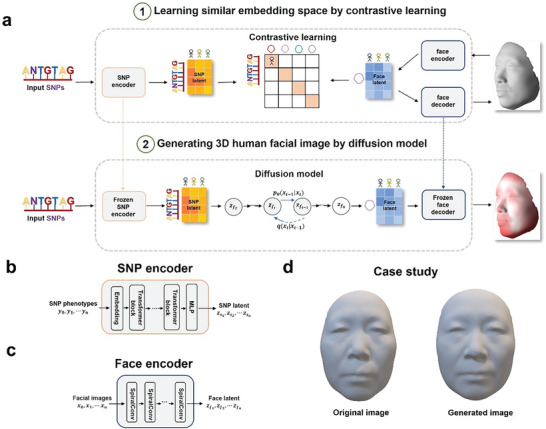
The overall framework of Difface. a) depicts the operation of Difface on a dataset comprising N SNP sets and their corresponding facial data. The initial phase of the model's training involves the merging of SNP‐face pairs into a unified representation space, laying the foundation for the generation phase. This entails the construction of a multimodal encoding space through the training of SNP and face encoders. The encoders enhance the cosine similarity between the correct SNP and face pair embeddings within each batch, while simultaneously reducing the similarity between the N × N incorrect pairs. Concurrently, a decoder is trained to generate 3D faces from face embeddings. Subsequently, an SNP embedding is introduced into a diffusion model, resulting in the generation of a face embedding. This face embedding is then utilized by the decoder to create the final image. It is noteworthy that the encoders and decoder remain fixed throughout the training of the diffusion model. b) Architecture of the SNP encoder. c) Architecture of the face encoder. d) A case study demonstrating the original and generated images.

We proposed Difface, a multi‐modality model designed to reconstruct 3D facial images from SNP phenotypes. Specifically, Difface employs a transformer^[^ ^31^ ^]^ and spiral convolution network^[^ ^32^ ^]^ to map high‐dimensional Single Nucleotide Polymorphisms (SNPs) and 3D point clouds onto a unified low‐dimensional feature space. At the same time, a spiral convolutional network was trained to generate facial images from the low‐dimensional feature space. During the training process a contrastive learning was used to construct the association between genetic and facial images in the low‐dimensional features, while during constructing process, Difface uses a diffusion network to generate 3D human facial images only from genetic features on each individual (**Figure** **1**).

### 2.2 Difface Enables the Alignment of DNA and 3D Facial Images

To evaluate whether Difface can accurately construct the association between DNA and 3D facial images, we employed Difface to analyze large‐scale 3D facial surface scans of 9674 samples collected from three independent cohorts in China (Methods). Using the conventional genome‐wide significance threshold (P = 5 × 10^−8^ ), we identified 7842 genome‐wide significant variants associated with 3D facial images. These variants were treated as input SNPs, constituting the input SNP vector of Difface.

Difface differs from Mahdi's^[^ ^23^ ^]^ and Sero's^[^ ^22^ ^]^ approaches by employing a unique implementation of contrastive learning. Unlike Mahdi's reliance on traditional regression models and Sero's use of basic machine learning without a focus on feature space alignment, Difface focuses on aligning genetic and phenotypic data in a low‐dimensional space, efficiently bridging complex genetic information with visible facial features. Difface enhances the specificity and sensitivity of feature extraction, providing a deeper understanding of the relationship between SNPs and facial features, and broadening the model's applicability to different datasets.

To comprehensively compare Difface with recently published methods (Mahdi's and Sero's), we trained Difface using the training dataset and then used two trained encoders to learn the low‐dimensional features of SNPs and facial images. Cosine similarity was employed to assess the alignment between facial images and SNP data. To validate the efficacy of our contrastive learning model, we utilized PCA^[^ ^33^ ^]^ and CCA^[^ ^34^ ^]^ to downscale SNP phenotypes and facial images. The comparison results demonstrated that Difface exhibited the highest alignment score among competing methods, indicating that contrastive learning is an efficient approach.

**Figure 2:**
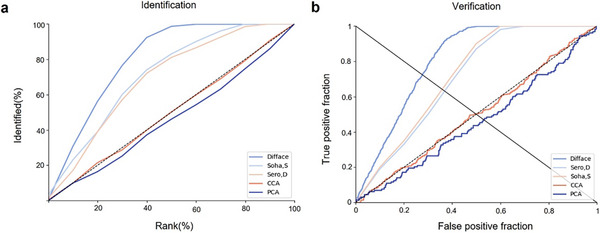
Performance comparison of Difface with other methods for identification and verification tasks. a) Identification performance: The y‐axis represents the percentage of correctly identified samples, while the x‐axis represents the rank percentage. Difface shows superior performance compared to Soha.S, Sero.D, CCA, and PCA. b) Verification performance: The y‐axis represents the true positive fraction, and the x‐axis represents the false positive fraction. The ROC curves indicate that Difface outperforms other methods in terms of verification accuracy.

Specifically, in terms of identification, Difface achieved a rank‐1 identification rate (R1) of 3.33%, a rank‐10 identification rate (R10) of 23.33%, and a rank‐20 identification rate (R20) of 42.53%, significantly outperforming other methods such as Mahdi's (R1: 2.48%, R10: 21.90%, R20: 38.49%) and Sero's (R1: 3.00%, R10: 23.03%, R20: 39.00%). In verification tasks, Difface also demonstrated superior performance with an equal error rate (EER) of 27.6% and an area under the curve (AUC) of 80.7%, compared to Mahdi's (EER: 35.7%, AUC: 72.1%) and Sero's (EER: 36.8%, AUC: 70.6%) (**Figure** **2**a,b) **Table** **1**.

**Table 1:** Average identification and verification results for different methods.

Method	EER	AUC	R1	R10	R20
Difface	27.6	80.7	3.33	23.33	42.53
Sero,D	35.7	72.1	2.48	21.90	38.49
Soha, S*	36.8	70.6	3.00	23.03	39.00
PCA	49.4	49.9	0	6.00	11.00
CCA	53.2	45.7	1.50	10.00	18.50

EER verification equal error rate, AUC verification area under the curve, R1 rank 1% identification rate, R10 rank 10% identification rate, R20 rank 20% identification rate.

These results indicate that contrastive learning enables Difface to not only capture complex genetic features but also differentiate between correct SNP‐face pairs and incorrect ones by pulling the correct pairs closer together in the feature space. This allows for more precise facial reconstructions compared to retrieval‐based or traditional methods. Difface, by utilizing advanced contrastive learning techniques, significantly improves the alignment and reconstruction of 3D facial images from DNA data, providing a more accurate and robust tool for forensic identification and personalized medicine.

### 2.3 Difface Enables the Generation of DNA and 3D Facial Images

**Figure 3:**
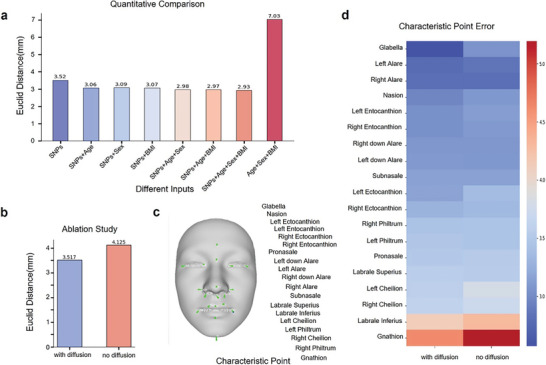
Euclidean Distance Comparison and Error Analysis for Facial Reconstruction Models. a) Quantitative comparison of different inputs on Euclidean distance (mm) between predicted and true facial landmarks. Inputs include SNPs, age, sex, BMI, and their combinations. b) Ablation study showing the impact of the diffusion model on Euclidean distance (mm). c) Visualization of characteristic points on the face used for error measurement. d) Heatmap showing the characteristic point error for models with and without diffusion. The color intensity represents the magnitude of the error, with red indicating higher error values.

To further assess the ability of Difface for reconstructing 3D facial images from single nucleotide polymorphisms (SNPs), we leveraged the Euclidean metric to evaluate performance by calculating the distance between the real and generated 3D point clouds. Our findings indicate that Difface, when leveraging only SNPs, achieved a mean error of 3.52 mm, which establishes a solid baseline for facial reconstruction fidelity. This result demonstrates that Difface can effectively reconstruct facial images from SNPs (**Figure** **3**a). We similarly evaluated the results using chamfer distance and RMSE (Figure S1, Supporting Information).

A detailed analysis of error metrics at critical facial landmarks showed that Difface consistently achieved significantly lower error distances, confirming its exceptional precision in reconstructing key facial features. The heatmap in Figure 3d illustrates that with the diffusion model, errors at specific facial points such as the glabella, nasion, and left/right alare were notably reduced, with errors mostly staying below 4.0 mm, whereas without the diffusion model, some points showed errors exceeding 4.5 mm.

In our evaluation of Difface, we assessed the contribution of the diffusion network to the model's performance. The diffusion technique has gained recognition for its effectiveness in generative tasks across various fields. The results, depicted in Figure 3c, show a significant enhancement in feature generation within the low‐dimensional space attributed to the diffusion network. Specifically, the mean Euclidean distance error with the diffusion network was 3.517 mm, compared to 4.125 mm without it, highlighting the crucial role of the diffusion network in achieving accurate feature generation.

Inspired by the flexible framework of Difface, we additionally incorporated SNP embeddings with more biological and demographic variables such as sex, age, and BMI. The comparison results showed that the addition of these variables systematically improved the quality of the reconstructed facial images. For instance, when combining SNPs with sex data, the mean error decreased to 3.06 mm, and further inclusion of age and BMI data reduced the error to 2.93 mm. Strikingly, when Difface operated without SNP data, relying solely on age, BMI, and sex, the mean error surged to 7.03 mm, reinforcing the critical role of genetic information in precise facial reconstruction. We also explored the impact of age as an additional variable. The quantitative analysis of these transitions revealed a change magnitude of 1.430 from youth to middle age, 1.689 from middle age to old age, and a total transformation of 2.415 from youth to old age. These values reflect the extent of facial structural change, such as the drooping of facial features and other age‐related changes, and are visually demonstrated in Figure S6 (Supporting Information). The progression from youth to old age is shown through the corresponding 3D reconstructions, illustrating how Difface captures age‐related morphological transformations.

These quantitative results not only validate the robustness of Difface in aligning and reconstructing 3D facial images from DNA data but also underscore its potential as a versatile tool in personalized medicine and forensic identification.

### 2.4 Difface Facilitates Individual Reconstruction of 3D Facial Images by Diffusion Process

We further demonstrated that Difface is able to individually reconstruct facial images with the diffusion process. In particular, we leveraged the latent features of the real and generated facial images by the trained encoder to predict the facial phenotypes. For each phenotype characteristics, we first trained each classifier by the training dataset, and then calculated the classification accuracy for the test dataset. Considering the pronounced hereditary influence on nasal morphology, our study stratified facial features into two principal categories: those associated with the nose and those about other facial regions. We conducted distinct classification tests for various features within these two categories.

**Figure 4:**
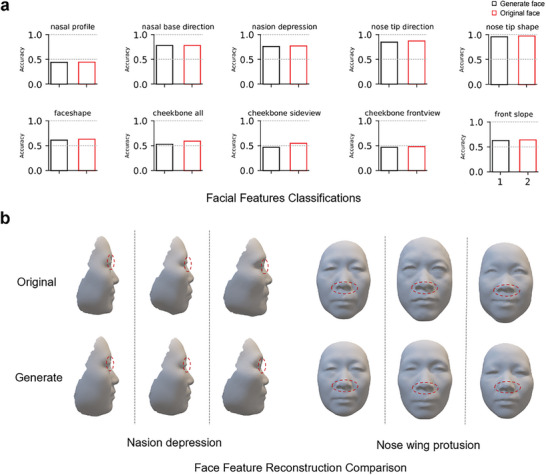
Comparison of Facial Feature Classification and Reconstruction Accuracy Using Difface and Ground Truth Data. a) Face feature classification results, Difface (SNPs), is the outcome of the classification task after utilizing Difface to produce faces from SNPs. Ground truth is the result of the original face for the classification task. b) Comparing the Difface restored face image with the real face image, the top is the real face image, and the bottom is the Difface restored face image.

The classification results, showcased in **Figure** **4**a, reveal that the correct classification rate for reconstructed faces is impressively close to that of real faces across multiple feature classifications. This result highlights the model's capability to precisely reproduce a broad range of facial features, demonstrating the strength and accuracy of Difface in capturing complex facial characteristics. We also present a direct comparison between original and generated 3D facial reconstructions, focusing on features such as nasion depression and nose wing protrusion. The aim is to demonstrate the model's capacity to mimic critical facial characteristics effectively.(Fig 4b).Similarly, We also compared the generated 3D faces with the original 3D faces and 2D facial photographs(Figure S2, Supporting Information).

In an innovative experimental setup designed to evaluate the efficacy of the Difface model in generating lifelike facial images, we conducted a blinded test involving 10 volunteers. Each participant was tasked with identifying the real face corresponding to a synthetically generated face from a lineup of real faces. To increase the challenge, we provided lineups of 5, 10, and 20 faces, which included the correct match alongside random real faces (**Table** **2**).

**Table 2:** Accuracy of Facial Recognition Across Different Lineup Sizes.

Lineup Size	Correct Identification Rate [%]
5 Choices	75.6
10 Choices	53.3
20 Choices	51.1

The experiment was structured to ascertain the likelihood of the participants correctly identifying the real face corresponding to the synthetic version at different lineup sizes. The results revealed a clear trend: as the number of choices increased, the accuracy of identification decreased, underscoring the challenges of facial recognition in larger lineups. The specific findings are summarized in the table below:

Furthermore, we leveraged the Determinantal Point Process (DPP) (a sophisticated method traditionally applied in subset selection problems with diversity constraints^[^ ^35^ ^]^) to quantify the diversity in the facial features generated by Difface. We calculated the DPP score for actual facial datasets and compared it with the DPP score of the average diversity derived from multiple random samplings of facial features. This allowed us to establish two robust baselines for the analysis. Additionally, we computed the DPP diversity for the facial images generated by Difface.

The results notably indicate that the DPP diversity score of the facial features generated by Difface is within a 5% margin of the diversity score calculated from the real facial data set. This proximity in diversity scores suggests a high level of variance in the features generated by Difface, closely mirroring the natural variability observed in human facial features. Furthermore, a threshold for desirable diversity at a DPP score that exceeds 95% of the real data's diversity score, which Difface consistently meets or surpasses. Consequently, it can be concluded that Difface successfully achieves a desirable level of diversity in its generated outcomes, effectively capturing the complex diversity in human facial features (**Table** **3**).

**Table 3:** Analysis Results of Data Diversity.

Data	DPP‐Diversity
Difface (SNPs)	0.9960
Real images	0.9999
Mean value	0.6012

### 2.5 Generative Effect Test for SNP Deletions

In practical applications, particularly in the field of forensics, it is often challenging to obtain a comprehensive set of single nucleotide polymorphisms (SNPs) from DNA samples. To assess the generative efficacy of Difface under conditions of SNP scarcity, evaluations were conducted with inputs ranging from 90% to 10% of the total SNP phenotypes. This approach was designed to simulate the constraints commonly encountered in forensic analyses.

**Figure 5:**
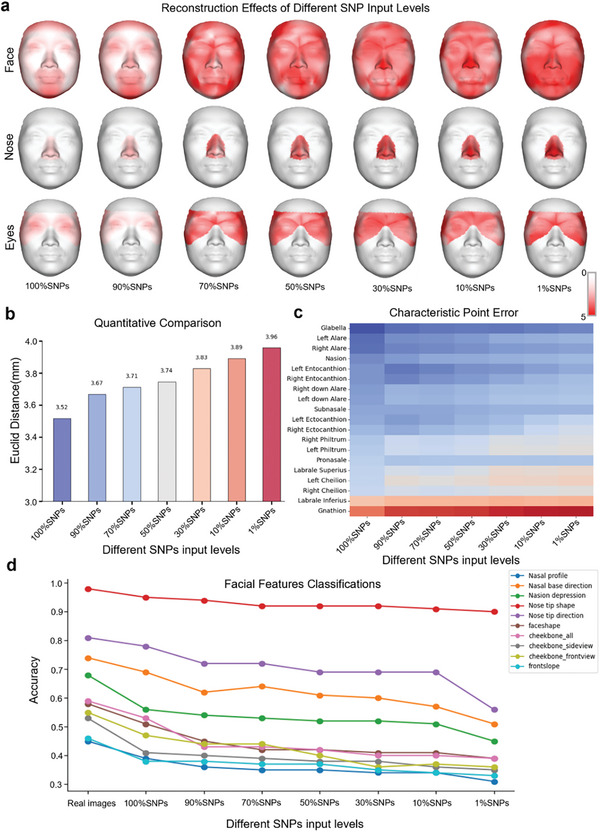
Impact of SNP Input Density on Facial Reconstruction Accuracy and Feature. a) Visual representation of facial reconstructions with different levels of SNP inputs, from 100% to 1%, illustrating the impact of SNP completeness on the visual accuracy of generated facial models. The first row is the whole face; the second row is the nose region; and the third row is the eye region. b) A bar graph shows the mean accuracy of facial reconstructions at different SNP densities, indicating a trend of decreasing accuracy with lower SNP percentages. c) A heat map displays the mean error rates for different facial regions across varying SNP densities, with darker shades indicating higher error values. d) Line graphs represent the stability of facial feature predictions (nasal profile, nasal base direction, nasal depression, nose shape, and nose tip direction) across different SNP input densities. Also, line graphs depict the accuracy of specific facial regions (face shape, cheekbone, cheekbone_altview, cheekbone_frontview, and frentislope) at different SNP input percentages.

The efficacy of facial reconstruction was evaluated across a spectrum of SNP completeness levels, ranging from 10% to 90%. Recent studies^[^ ^19^ ^]^ have demonstrated that the nose exhibits the strongest genetic associations among facial features, with 107 genetic variants identified surpassing other regions of the face. The distinctive nose shape commonly observed in East Asians is largely attributed to local adaptations to environmental factors and genetic drift. While natural selection did play a role in shaping this trait, the selective pressures were less intense compared to those experienced by European populations. Specific genetic variants in East Asians contributed to the evolution of their characteristic flatter and broader nose shape, despite the underlying biological processes being similar across different populations. Given these findings, we focused our analysis on how the nose region changes as the SNP input decreases. As illustrated in **Figure** **5**, the nose, particularly the nasal profile and tip shape, remains relatively resilient at higher SNP levels but starts to show increased error as the SNP input drops below 70%. We also analyzed the variation in error across the face and eye region, giving us a broader understanding of the effect of SNPs on different features. The Euclidean distance—which is used to quantify the discrepancy between the reconstructed and actual facial structures—increases as the level of input SNPs decreases. With complete SNP input (100%), the error is minimized at 3.517 mm. Conversely, as the proportion of SNP inputs is reduced to 10%, the error increases significantly to 3.957 mm, thereby demonstrating a marked decline in reconstruction accuracy. The heatmap provides a detailed analysis of the errors at various characteristic facial points at different SNP levels. As the SNP completeness diminishes, the error amplification at points such as the nasion, subnasale, and cheilion increases. Furthermore, the capacity to accurately categorize specific facial features, such as the nasal profile, nose tip shape, and cheekbone appearance, is diminished as the SNP input is reduced. Notably, the classification accuracy for certain features, such as the nasal profile, remains relatively high even at lower SNP levels. This suggests that certain facial aspects are more resilient to SNP scarcity than others. Extended data 3 shows more details of the change in error for generating surfaces as the number of SNPs decreases.

Despite the retention of some facial features with limited SNP data, a critical threshold was observed. When the completeness of the SNP data fell below 70% (equating to fewer than 5489 SNPs from the full set of 7842), the reconstructions began to lose individual specificity and converged toward a more generic facial structure. This observation highlights the necessity of a sufficiently comprehensive SNP dataset to preserve the distinctive, individualised characteristics essential for forensic applications. It is of paramount importance to strike a balance between the robustness of the model and the availability of genetic data in order to ensure that facial reconstructions remain both accurate and person‐specific. This balance is of vital importance for the enhancement of the utility and reliability of SNP‐based facial reconstruction in real‐world forensic settings.

### 2.6 Enhancing Model Interpretability in Facial Reconstruction with SHAP and GWAS

In order to further refine and elucidate the mechanism behind the Difface model, we employed SHapley Additive exPlanations^[^ ^36^ ^]^ (SHAP), a method based on cooperative game theory. SHAP assigns quantitative values to individual SNP features, allowing for the precise determination of how each feature influences the model's predictions. By decomposing a prediction into the contributions of specific SNPs, SHAP enables us to better understand the role of each genetic marker in shaping facial morphology.

This approach was complemented by an enrichment analysis using genome‐wide association studies (GWAS), focusing on SNPs with high SHAP values and significant p‐values (*p* < 0.05). Through this dual analysis, we identified key SNPs that not only play a prominent role in the model's output but also have strong genetic associations with facial structure. For instance, SNPs like nasal_base_direction and nasal_root_height consistently exhibited low hypergeometric p‐values across multiple sample sizes, underscoring their relevance in both SHAP and GWAS analyses.

**Figure 6:**
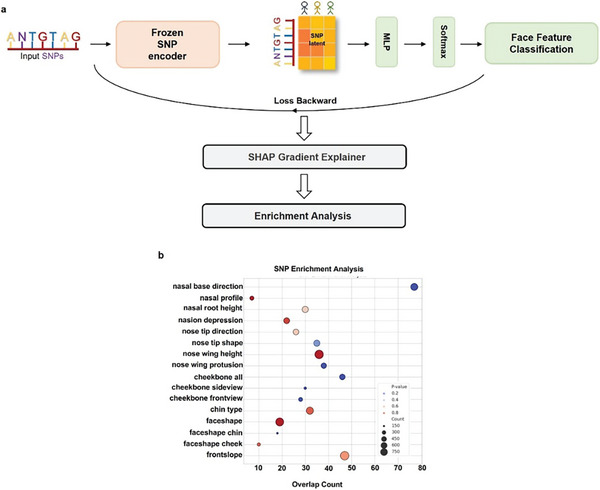
a) The diagram illustrates the Difface model's workflow, starting from the input of SNPs through to facial feature classification. The process includes the transformation of SNPs by a Frozen SNP encoder, SNP latent feature extraction, feature classification, and the back‐propagation of losses for SHAP value calculation. The workflow concludes with an enrichment analysis to correlate SNP effects with biological processes. b) SNP Enrichment Analysis: This graph shows the overlap count between SNPs identified by SHAP values and those found significant in GWAS studies for various facial features. Each point represents a facial feature with its corresponding overlap count, indicating the statistical significance (*p*‐value) through color coding.

As shown in **Figure** **6**a, the workflow begins with input SNPs processed through the frozen SNP encoder, followed by a multi‐layer perceptron (MLP) and softmax classifier to predict facial feature classifications. SHAP values are then calculated via backward loss propagation to assess the impact of individual SNPs on the model's predictions. The SNPs identified through SHAP are further analyzed for biological significance through enrichment analysis.

The results, presented in Figure 6b, demonstrate a high degree of overlap between SHAP‐determined SNP importance and SNPs identified in GWAS, confirming the robustness and interpretability of the model. SNPs relevant to features like nasal profile and cheekbone sideview were highlighted in both SHAP and GWAS results, providing strong evidence that the genetic associations are consistent with known biological influences.

Moreover, the analysis extended beyond statistical significance to explore the biological implications of these findings. Gene Ontology (GO) enrichment analysis revealed that many of these SNPs are involved in processes critical to facial development, such as “regulation of epithelial to mesenchymal transition” and “morphogenesis of embryonic epithelium.” These processes are vital to facial structure formation, influencing both bone and muscle development, as well as vascularization and symmetry. Notably, pathways like “skeletal system development” and “muscle organ development” further underscore the role of these SNPs in defining the face's structural framework. (Figure S4, Supporting Information)

This combination of SHAP and GWAS highlights the interpretability of the Difface model by showing how the SNPs identified as crucial by SHAP analysis are also statistically significant in genetic studies. The congruence between these two methods provides compelling evidence that Difface not only reconstructs facial features with high accuracy but does so in a manner consistent with established genetic influences on facial morphology.

By integrating SHAP and GWAS into our analysis, we have demonstrated that the Difface model is both interpretable and biologically grounded. These findings pave the way for future research into the genetic underpinnings of facial architecture, offering potential targets for exploring the effects of genetic variations on facial structure.

## 3 Discussion

This study has introduced Difface, a de novo multi‐modality model to reconstruct 3D facial images from DNA with remarkable precision, by a generative diffusion process and a contrastive learning scheme. Through comprehensive analysis and SNP‐FACE matching tasks, Difface demonstrated superior performance in generating accurate facial reconstructions from genetic data. In particularly, Difface could generate/predict 3D facial images of individuals solely from their DNA data at various future ages. Notably, the model's integration of transformer networks with spiral convolution and diffusion networks has set a new benchmark in the fidelity of generated images to their real images, as evidenced by its outstanding accuracy in critical facial landmarks and diverse facial feature reproduction.

Difface's novel approach, combining advanced neural network architectures, significantly outperforms existing models in genetic‐to‐phenotypic facial reconstruction. This superiority is attributed to its unique contrastive learning method of aligning high‐dimensional SNP data with 3D facial point clouds in a unified low‐dimensional feature space, a process further enhanced by adopting diffusion networks for phenotypic characteristic generation. Such advancements contribute to the model's exceptional precision and ability to capture the subtle genetic variations influencing facial morphology, a feat less pronounced in previous methodologies.

Despite Difface's demonstrated strengths, there remain directions for improvement. Addressing these limitations will require a focused effort to increase the model's robustness and adaptability to diverse datasets. Future research should aim to incorporating variables like age and BMI would allow Difface to simulate age‐related changes, enabling the generation of facial images at different life stages an application that holds significant potential in both forensic science and medical diagnostics. Similarly, BMI could help the model account for variations in body composition, improving its ability to generate accurate facial reconstructions across a range of body types.

While our study focused on a genetically homogeneous East Asian cohort, the underlying structure of the Difface model is flexible enough to be applied to different ethnic groups. By expanding our dataset to include individuals from a wide range of ethnic backgrounds, Difface has the potential to learn the specific relationships between SNPs and facial features in various populations. This would enable the model to accurately generate corresponding facial images based on the SNP input from individuals of different ethnicities.Future studies should focus on this crucial extension by testing and adapting the model to encompass more genetically diverse populations. Validating Difface with datasets from multiple ethnic groups and exploring whether additional genetic loci are necessary for certain facial features will be key steps to ensuring that the model generalizes effectively across diverse populations. This broader application will not only enhance the model's robustness but also significantly increase its utility in real‐world forensic investigations and personalized medicine on a global scale.

However, this innovative technology brings forth critical ethical and legal challenges, particularly concerning genomic privacy. The ability to associate de‐identified genomic data with phenotypic characteristics raises substantial risks, including potential re‐identification, unauthorized data usage, and complex legal issues surrounding data ownership. By enabling the prediction of physical traits from genetic information, DNA phenotyping intensifies concerns about the misuse of sensitive personal data, which could lead to genetic‐based discrimination and privacy infringements. It is therefore essential for researchers, policymakers, and industry leaders to engage in proactive dialogue to address these ethical and legal complexities.

Beyond academic exploration, the societal impacts of DNA phenotyping are extensive, touching on sensitive fields such as forensic science, healthcare, and insurance. In forensic applications, while the technology has potential benefits in identifying individuals from genetic material, improper use could result in significant harm, including wrongful convictions and racial or ethnic profiling. In healthcare and insurance, there is the risk of genetic information being used to discriminate against individuals based on perceived health risks.

To mitigate these risks, we emphasize the urgent need for comprehensive interdisciplinary analysis. Establishing clear, enforceable ethical frameworks and guidelines is paramount to ensuring that genomic research advances responsibly, safeguarding individual privacy and rights while harnessing the potential benefits of this technology. Such frameworks will help balance scientific progress with societal responsibility, creating a foundation for ethical and transparent application of DNA phenotyping across various fields.

## 4 Experimental Section

### Difface

We proposed Difface, a multi‐modality model designed to reconstruct 3D facial images from SNPs with a generative diffusion process and a contrastive learning scheme. Specifically, Difface separately employs a transformer^[^ ^31^ ^]^ and spiral convolution network^[^ ^32^ ^]^ to map high‐dimensional SNPs (Y) and 3D point clouds (X) into the same low‐dimensional feature spaces (*z_f_* and *z_s_*) while ensuring the paired SNPs and facial images are embedded nearly compared to unpairs by contrastive learning. Subsequently, Difface uses a diffusion model to transform SNP embeddings *z_s_* into facial image embeddings *z_f_* conditioned on SNPs *y*. Finally, 3D facial images are generatively reconstructed from facial image embeddings *z_f_* by a decoder *P*(*x*|*z_f_*,*y*), as shown in Figure 1.

### Learning Related Features Between SNPs and 3D Point Cloud by Contrastive Learning

Our contrastive learning approach is designed to map high‐dimensional SNP data and 3D facial point clouds into a unified low‐dimensional feature space, leveraging a cosine similarity metric to ensure accurate alignment of true SNP‐face pairs while reducing the similarity of incorrect pairings. To achieve this goal, we have designed a face encoder and a SNP encoder, as well as a decoder to generate faces from embeddings, which further constrain the potential space. The spiral convolution network was chosen to capture localized spatial features in 3D point clouds effectively. Compared to traditional convolutions, spiral convolution allows for better handling of the non‐grid structure of 3D data, such as facial point clouds, by maintaining the spatial relationships between points. The specific model is described as follows:

The face encoder design was inspired by SpiralConv++^[^ ^32^ ^]^ which designs a spiral neighbor as:

(1) $$0-\mathrm{ring}\;\left(v\right)=v$$

(2) $$k-\mathrm{disk}\;\left(v\right)=\cup_{i=0,\text{…},k}\;i-\mathrm{ring}\left(v\right)$$

(3) $$\left(k+1\right)-\mathrm{ring}\;\left(v\right)=N\left(k-\mathrm{ring}\left(v\right)\right)\setminus k-\mathrm{disk}\left(v\right)$$

where *n*(*v*) is the set of all vertices adjacent to any vertex in set *V*, *V* corresponds to the vertices a point cloud network, and the length of the Spiral is represented as *l*. Hence, *S*(*v*, *l*) is an ordered set of k‐rings connected by *l* vertices.

The spiral convolution operator for node *i* is defined as follows:

(4) $$i_{\left(k\right)}^{x}=\gamma^{\left(k\right)}\;\left(\sum_{j\in S\left(i,l\right)}j_{\left(k-1\right)}^{x}\right)$$

In this context, γ denotes MLPs. The encoder's structure is: 3 × {Conv(32)→ Pool(4)}→ {Conv(64) → Pool(4)} →FC(128), with RELU activation function after each Conv layer.

The structure of the decoder is the reversed order of the encoder with the replacement of pooling layers to unpooling layers. Note that one more convolutional layer with the output dimensional of 3 should be added to the end of the decoder to reconstruct 3D shape coordinates.

A transformer block is utilized to construct the SNP encoder. The model architecture was determined based on the original Transformer^[^ ^31^ ^]^ with a model dimension of 128, a feed‐forward layer dimension of 512, and 2 layers in the encoder. ReLU activation was used, and the encoder had a dropout ratio of 0.1. The 7842 SNP phenotypes were embedded as a sequence and then input into the transformer block after dimensionality reduction by a Multilayer Perceptron (MLP).

We use *L*_1_ loss for the generation loss *Loss_mesh_* and cross‐entropy is used as the loss *Loss_con_* for contrastive learning. Formally, we have

(5) $$\mathrm{Los}s_{\mathrm{mesh}}=\frac{1}{N}\;\sum_{i=1}^{N}\left|{\hat{x}}_{i}-x_{i}\right|$$

(6) $$\mathrm{Logi}t_{1}=\alpha \left({z_{f}}_{i}\times {s_{i}}_{T}^{z}\right)$$

(7) $$\mathrm{Logi}t_{2}=\alpha \left({z_{s}}_{i}\times {f_{i}}_{T}^{z}\right)$$

(8) $$\mathrm{Los}s_{\mathrm{con}}=\frac{l_{CE}\left(\mathrm{Logit}1,\mathrm{labels}\right)+l_{CE}\left(\mathrm{Logit}2,\mathrm{labels}\right)}{2}\;$$

where α is a hyperparameter, and *z_f_*,*z_s_* are face embeddings and SNP embeddings, respectively. *l_CE_* is the cross‐entropy loss, and *labels* is a vector just like [0, 1, 2, ⋅⋅⋅, *N* − 1], where *N* is the batch size.

Thus, our overall loss function is

(9) $$\mathrm{Los}s_{\mathrm{ALL}}=\beta_{1}\;\mathrm{Los}s_{\mathrm{con}}+\beta_{2}\mathrm{Los}s_{\mathrm{mesh}}$$

where β_1_,β_2_ are hyperparameters.

### Reconstructing 3D Facial Images from SNPs by Diffusion Model

We trained an encoder to generate SNP embeddings and facial embeddings, along with a decoder for generating faces from facial embeddings. However, we require a prior model to generate facial embeddings from SNP embeddings. To achieve this, we employ a diffusion model^[^ ^37^, ^38^, ^39^ ^]^ in this context.

The diffusion model operates through a two‐step process: the forward diffusion and the reverse denoising. During the forward diffusion process, we introduce Gaussian noise into the facial embeddings ${z_{f}}_{0}∼q\left(z_{f}\right)$ over *T* steps. To accomplish this, a series of hyperparameters representing Gaussian distribution variances $t=1_{T}^{\left\{\beta_{t}\in \;\left(0,1\right)\right\}}$ are required. The forward process can be viewed as a Markov process since each time step *t* only depends on the previous time step *t* − 1. So, it can also be regarded as a Markov process.

(10) $$\begin{array}{ccc} q({z_{f}}_{t}|{z_{f}}_{t-1}) & = & N\left({z_{f}}_{t};\sqrt{1-\beta_{t}}{z_{f}}_{t-1},\beta_{t}I\right),q\left(q\left({z_{f}}_{1:T}|{z_{f}}_{t-1}\right)|x_{0}\right)\\ & = & \prod_{t=1}^{T}q\left({z_{f}}_{t}|{z_{f}}_{t-1}\right) \end{array}$$

In this process, as *t* increases, *z_f_*_*t*_ becomes progressively closer to pure noise. When *T* → ∞, *z_f_*_*T*_ becomes entirely Gaussian noise. If we consider the forward process as the noise introduction process, then the reverse process is the denoising diffusion process of the diffusion model. If we could obtain the reverse distribution step by step *q*(*z_f_*_*t* − 1_|*z_f_*_*t*_), we could recover the original distribution *x*_0_ from the fully standard Gaussian distribution ${z_{f}}_{T}∼N\left(0,I\right)$. However, inferring *q*(*z_f_*_*t* − 1_|*z_f_*_*t*_) is not straightforward. Therefore, we use a deep learning model to predict a reverse distribution *p*_θ_.

(11) $$p_{\theta }\;\left(X_{0:T}\right)=p\left({z_{f}}_{T}\right)\prod_{t=1}^{T}p_{\theta }\left({z_{f}}_{t-1}|{z_{f}}_{t}\right)$$

(12) $$p_{\theta }\left({z_{f}}_{t-1}|{z_{f}}_{t}\right)=N\left({z_{f}}_{t-1};\mu_{\theta }\left({z_{f}}_{t},t\right),\sum_{\theta }\left({z_{f}}_{t},t\right)\right)$$

While we may not have access to the reverse distribution *q*(*z_f_*_*t* − 1_|*z_f_*_*t*_), if we know *x*_0_, we can indeed use Bayes' theorem to obtain *q*(*z_f_*_*t* − 1_|*z_f_*_*t*_,*z_f_*_0_).

(13) $$q\left({z_{f}}_{t-1}|{z_{f}}_{t},{z_{f}}_{0}\right)=N\left({z_{f}}_{t-1};\tilde{\mu }\left({z_{f}}_{t},{z_{f}}_{0}\right),\tilde{\beta_{t}}I\right)$$

To achieve the generation of facial images from SNPs, SNP embeddings were utilized as a conditional bootstrap diffusion model to generate facial embeddings. The forward process of the conditional diffusion model is identical to that of the unconditional diffusion model. Hence, the joint probability of the backward process is

(14) $$p_{\theta }\;\left(X_{0:T}|y\right)=p\left({z_{f}}_{T}\right)\prod_{t=1}^{T}p_{\theta }\left({z_{f}}_{t-1}|{z_{f}}_{t},y\right)$$

We find it better to train our model to predict the unnoised *z_f_*_*T*_ directly, and thus use a mean‐squared error loss on this prediction:

(15) $$L_{\mathrm{diffusion}}=E_{t∼\left[1,T\right],i_{\left(t\right)}^{z_{f}}∼q_{t}}\;\left[∥f_{\theta }\left(i_{\left(t\right)}^{z_{f}},t,y\right)-{z_{f}}_{t}{∥}^{2}\right]$$

### Model Architecture and Components

The architecture of the Difface model is composed of several key components that work together to achieve high performance in reconstructing facial features from genetic data. At the core of the model is the integration of convolutional and transformer‐based networks, enabling effective feature extraction and the modeling of complex spatial relationships.

The face encoder employs a spiral convolutional network with a sequence length of [9, 9, 9, 9] and a dilation factor of [1, 1, 1, 1], allowing it to capture localized spatial features while expanding the receptive field without loss of resolution. Additionally, the SNP encoder consists of two transformer layers, each with a model dimension of 128, an 8‐headed self‐attention mechanism, and a feed‐forward network with a hidden dimension of 512. These layers incorporate a dropout rate of 0.1 and apply layer normalization with an epsilon value of 1e‐5, ensuring stable training and robust generalization.

The diffusion prior model is designed to predict the starting state without conditional dropout, operating with an hidden embedding dimension of 128 and 1 hidden channel. It features a 4‐layer depth and an attention mechanism with 4 heads, each with a head dimension of 64. The feed‐forward network has an expansion multiplier of 2, with a 0.1 dropout rate applied to both the attention and feed‐forward layers. The diffusion model operates over 1000 timesteps, allowing the model to handle input data directly and reconstruct facial features with high precision.

### Optimization and Parameters

To ensure robust performance and improve learning efficiency, we employed several optimization strategies in training the Difface model, including gradient descent algorithms and dynamic learning rate scheduling.

The Adam optimizer was selected as the primary optimization method. Different components of the model required tailored configurations. For the core model parameters, we utilized a learning rate of 7.3×10⁻⁶ and a weight decay of 0.30, preventing overfitting while enhancing generalization capabilities. For the decoder, which plays a key role in reconstructing 3D facial images from learned embeddings, a higher learning rate of 3.2×10⁻⁵ and a lower weight decay of 0.001 were applied. To further refine the training process, we incorporated a StepLR scheduler that dynamically adjusted the learning rate during training. The learning rate was reduced by a factor of 0.99 after each epoch, enabling a gradual decrease that fine‐tuned the model weights.

The diffusion prior model was trained with a learning rate of 0.31 × 10⁻⁴ and a weight decay of 0.752. A maximum gradient norm of 0.5 was enforced to prevent gradient explosion, ensuring stable convergence throughout the process. Additionally, an exponential moving average (EMA) was implemented with a beta value of 0.98, and updates were applied every 1000 steps, with EMA updates occurring every 100 steps to smooth the learning process and stabilize model weights.

We utilized a dataset of 9674 samples, each containing 3D facial images, SNP data, age, BMI, and other relevant information. The dataset was randomly split into training and testing sets, with 80% allocated for training and 20% for testing. The model was trained using four NVIDIA 3080Ti GPUs, with a total training time of ≈48 h.

### Quantification of Diversity of the Learned SNP Features

We measure this diversity using a metric based on the determinant of a kernel matrix:

(16) $$\mathrm{dpp}\;\mathrm{diversity}=\det\left(K\right)$$

where $K_{i,j}=\frac{1}{1+\mathrm{dist}(c_{i},c_{j})}$ and dist(*c_i_*,*c_j_*) represents the distance metric between real faces and generated faces. In practice, to avoid ill‐conditioned determinants, we introduce small random perturbations to the diagonal elements when computing the determinant.

### Evaluating Feature Importance

The Difface model employs a component known as the GradientExplainer to leverage SNP phenotypes and their corresponding facial images within the analytical framework. This tool is designed for use with models that incorporate gradient data in order to elucidate the relationship between input variables and output responses. By capturing and analyzing the gradient signals, the GradientExplainer identifies the extent to which the model's predictions are sensitive to variations in SNP inputs. The resulting SHAP values for each SNP provide a direct metric of their influence, offering a detailed and quantitative understanding of the genetic determinants affecting facial structure.

### SNPs Enrichment Analysis

We conducted two distinct enrichment analyses to better understand the genetic associations of SNPs identified through the Difface model:

Analysis of Top 200 SNPs: For each phenotype related to somatic anthropology, we selected the top 200 SNPs that showed the strongest correlation based on SHAP values. These SNPs were filtered to retain independent loci, which were then subjected to genetic annotation and subsequent enrichment analysis. This process aimed to identify biological pathways and processes that are significantly influenced by these SNPs.

Analysis of Top 500 SNPs: In this analysis, we selected the top 500 SNPs with the highest correlation, as determined by SHAP values.These SNPs were analyzed for enrichment against genome‐wide association studies (GWAS), focusing on those with a significance threshold of *p*‐value < 0.05. This allowed us to identify SNPs that are not only strongly correlated with the phenotypes but also statistically significant in broader genetic studies.

### Datasets and Processing Ethics Statement Sample and Recruitment Details

Ethics statement. All participants provided written informed consent, and all study protocols were approved by the institutional review boards of the pertinent research institutions. The National Survey of Physical Traits (NSPT) is a subproject of The National Science & Technology Basic Research Project approved by the Ethics Committee of Human Genetic Resources of School of Life Sciences, Fudan University, Shanghai (14117). The Northern Han Chinese (NHC) cohort was approved by the Ethics Committee of Human Genetic Resources at the Shanghai Institute of Life Sciences, Chinese Academy of Sciences (ER‐SIBS‐261410‐A1801). The Taizhou Longitudinal Study (TZL) was approved by the Ethics Committee of Human Genetic Resources at the Shanghai Institute of life Sciences, Chinese Academy of Sciences (ER‐SIBS‐261410). Written informed consent was granted to each participant before enrollment in the study. We confirm that our research is compliant with the Guidance of the Ministry of Science and Technology (MOST) for the Review and Approval of Human Genetic Resources.

The samples in this study were collected from three independent cohorts, the NSPT cohort (n = 3322), the NHC cohort (n = 4767) and the TZL cohort (n = 2881). For the NSPT sample, individuals were recruited in three Chinese cities: Nanning, Guangxi province (n = 1326); Taizhou, Jiangsu Province (n = 986); Zhengzhou, Henan Province (n = 1010). In the NHC cohort, participants were recruited in Tangshan, Hebei province.

### 3D Image Acquisition, Registration, and Quality Control

The 3D images of all individuals in the three cohorts were captured and acquired using the 3dMDface (3dMD) camera system. Participants were asked to close their mouth, open their eyes and hold faces with a neutral expression when capturing. The 3D surface images were registered using MeshMonk (v.0.0.6)^[^ ^40^ ^]^ in MATLAB 2018a. This performed a homologous configuration of 7906 spatially dense landmarks, allowing the 3D image data to be standardized. We performed generalized procrustes analysis (GPA) and symmetrization, then investigated every mapped image manually and identified outlier images. Any 3D facial images with poor quality were removed or reprocessed, with details available in the Supplementary Note. As a result, 6968 (n = 4089 in the NHC cohort, n = 2879 in the NSPT cohort) and 2706 unrelated individuals with good quality 3D images in the discovery and replication dataset were used for further analysis.

## Conflict of Interest

The authors declare no conflict of interest.

## Supporting information



Supporting Information

## Data Availability

The data that support the findings of this study are available on request from the corresponding author. The data are not publicly available due to privacy or ethical restrictions.
