# Supplementary material for: De Novo Reconstruction of 3D Human Facial Images from DNA Sequence
Source: Adv Sci (Weinh). 2025 May 7;12(29):2414507. doi: 10.1002/advs.202414507 (PMC12362825; doi:10.1002/advs.202414507)
Supplement: Supplementary file 1 — Supporting Information [file ADVS-12-2414507-s001.docx]

**De novo reconstruction of 3D human facial images**

**from DNA sequence**

Mingqi Jiao^1^*, Jiarui Li^2^*, Bingxu Zhong^1^, Siyuan Du^2,3^, Shuning Li^1^, Manfei Zhang^3^, Qibin Zhang^1^, Zhongming Liang^1^; Fan Liu^4^ ✉, Chunman Zuo^5^ ✉, Sijia Wang^2,6,7^✉, Luonan Chen^1,8,9^ ✉

^1^ Key Laboratory of Systems Health Science of Zhejiang Province, Hangzhou Institute for Advanced Study, University of Chinese Academy of Sciences, Chinese Academy of Sciences, Hangzhou 310024, China.

^2^ CAS Key Laboratory of Computational Biology, Shanghai Institute of Nutrition and Health, University of Chinese Academy of Sciences, Chinese Academy of Sciences, Shanghai, China.

^3^ Bio-X Institutes, Key Laboratory for the Genetics of Developmental and Neuropsychiatric Disorders, Ministry of Education, Shanghai Jiao Tong University, Shanghai 200030, China.

^4^ Naif Arab University for Security Sciences, Riyadh, Saudi Arabia

^5^ Institute of Artificial Intelligence, Donghua University, Shanghai 201620, China

^6^ Taizhou Institute of Health Sciences, Fudan University, Taizhou, Jiangsu, China.

^7^ Center for Excellence in Animal Evolution and Genetics, Chinese Academy of Sciences, Kunming, China.

^8^ Key Laboratory of Systems Biology, Shanghai Institute of Biochemistry and Cell Biology, Center for Excellence in Molecular Cell Science, Chinese Academy of Sciences, Shanghai 200031, China.

^9^ Guangdong Institute of Intelligence Science and Technology, Hengqin, Zhuhai, Guangdong 519031, China.


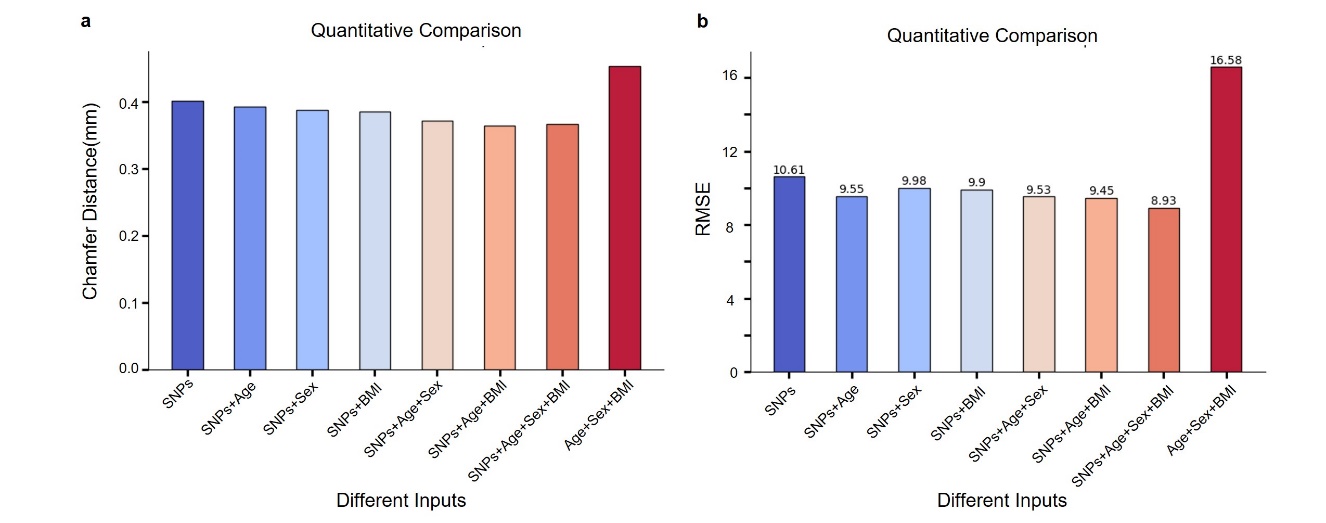
**Figure S1.** **Quantitative Comparison of Facial Reconstruction Accuracy Based on Different Input Factors** (a) This panel shows a quantitative comparison using chamfer distance metrics to evaluate the reconstruction accuracy of facial features with different input combinations. (b) Root Mean Square Error (RMSE) values for facial reconstruction using different input configurations.


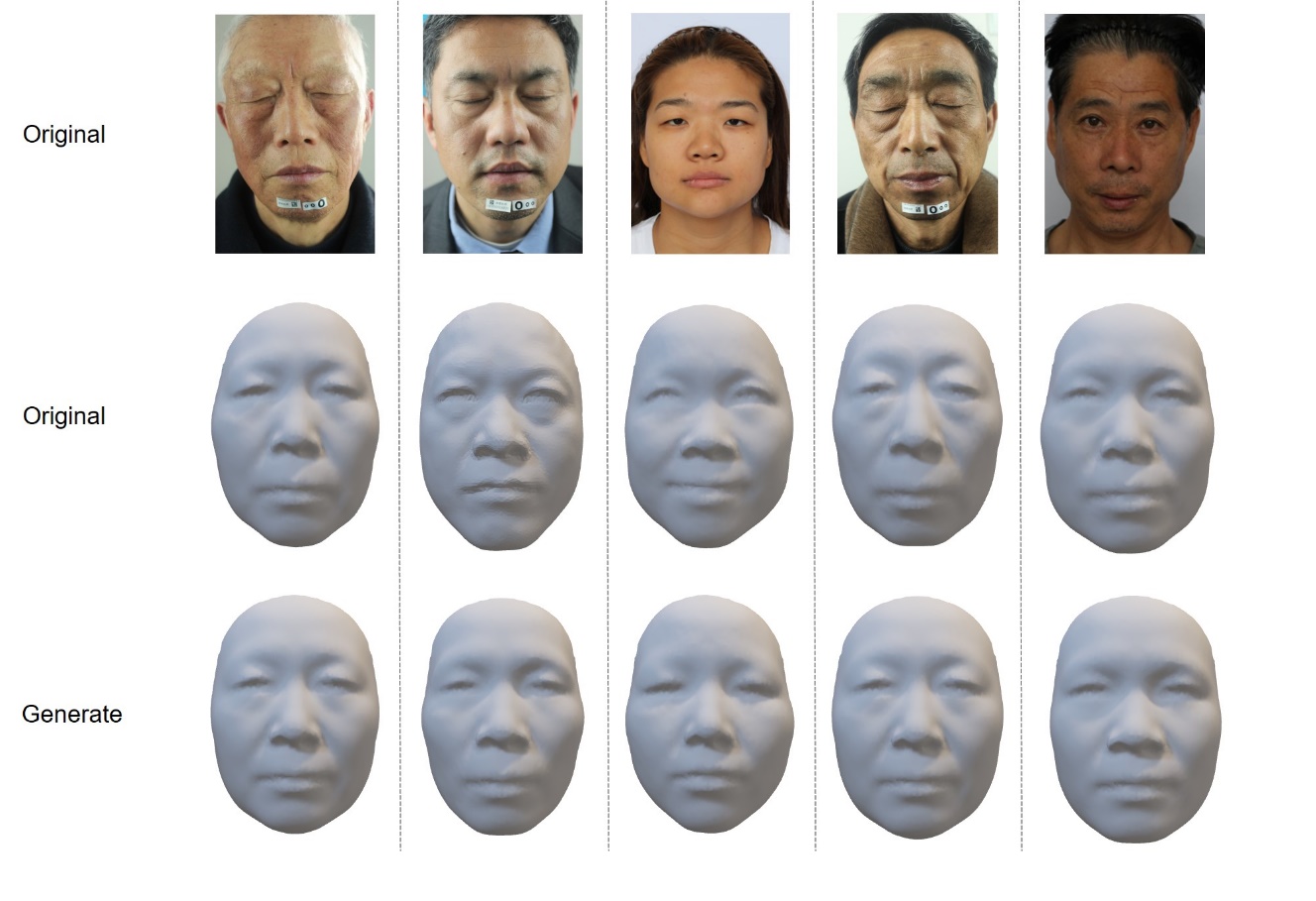


**Figure S2.** **Facial Reconstruction Fidelity Assessment.** This figure shows the fidelity of the facial reconstructions generated by the Difface model compared to the original human subjects. The top row shows original photographs of five individuals. Below each original photograph are two rows; the middle row shows the 3D scans of the original faces, while the bottom row shows the corresponding 3D face reconstructions generated by Difface. This visual comparison highlights the model's ability to capture distinct facial features and allows for an assessment of the accuracy and realism of the synthesized facial reconstructions across different ethnic and age groups.


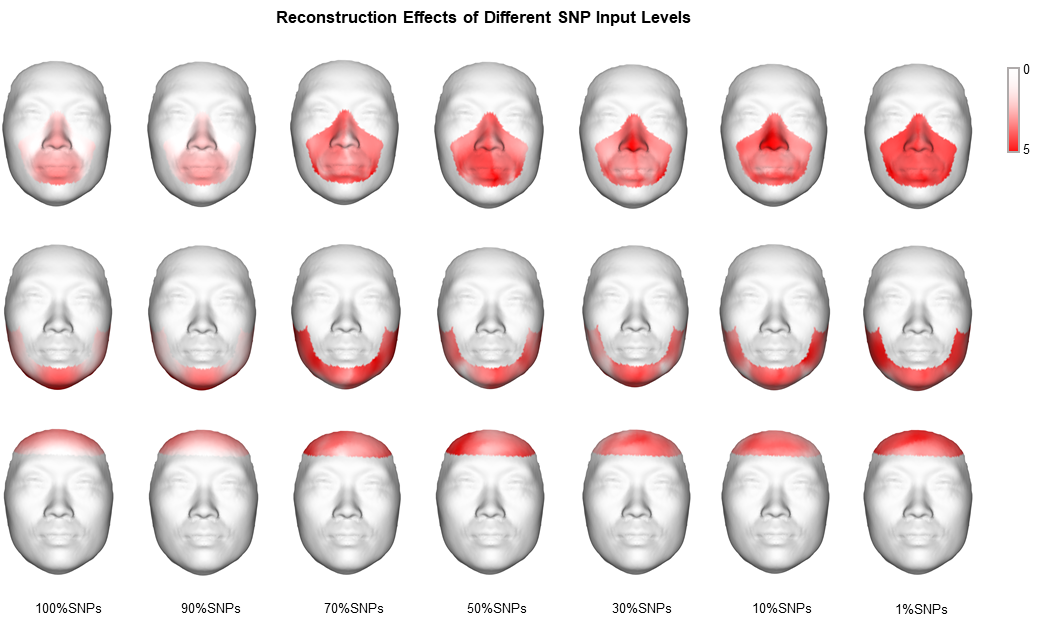


**Figure S3.** **Impact of SNP Input Levels on Facial Reconstruction Accuracy** **on Some Facial Regions.** This figure showcases the facial reconstruction effects on various regions except the whole face, nose region, and eye region under various SNP input levels, the color intensity on each facial model highlights areas of high reconstruction error, with darker red indicating higher error levels. This visualization effectively demonstrates how the quantity of input SNPs influences the accuracy and detail of the generated facial structures. Note that the whole face, nose region, and eye region are illustrated in Figure 3a.

**
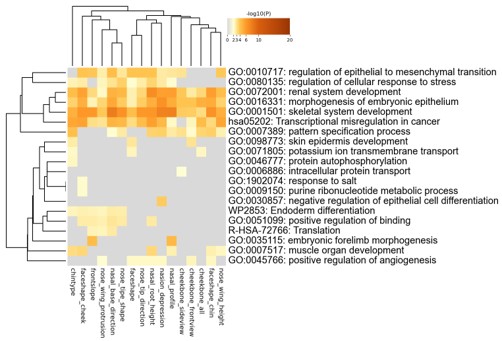
**

**Figure S4. Gene Ontology (GO) Enrichment Analysis of SNP Associations.** This heatmap and dendrogram illustrate the GO term enrichment analysis based on SNPs associated with specific facial features. Each cell in the heatmap corresponds to a specific GO term related to biological processes, with color intensity indicating the -log10(p-value), thus highlighting the significance of the enrichment. The dendrogram clusters similar GO terms, showing relationships and commonalities in biological functions potentially influenced by the identified SNPs. Notable terms include "regulation of epithelial to mesenchymal transition" and "muscle organ development," which are critical for facial development and morphology.


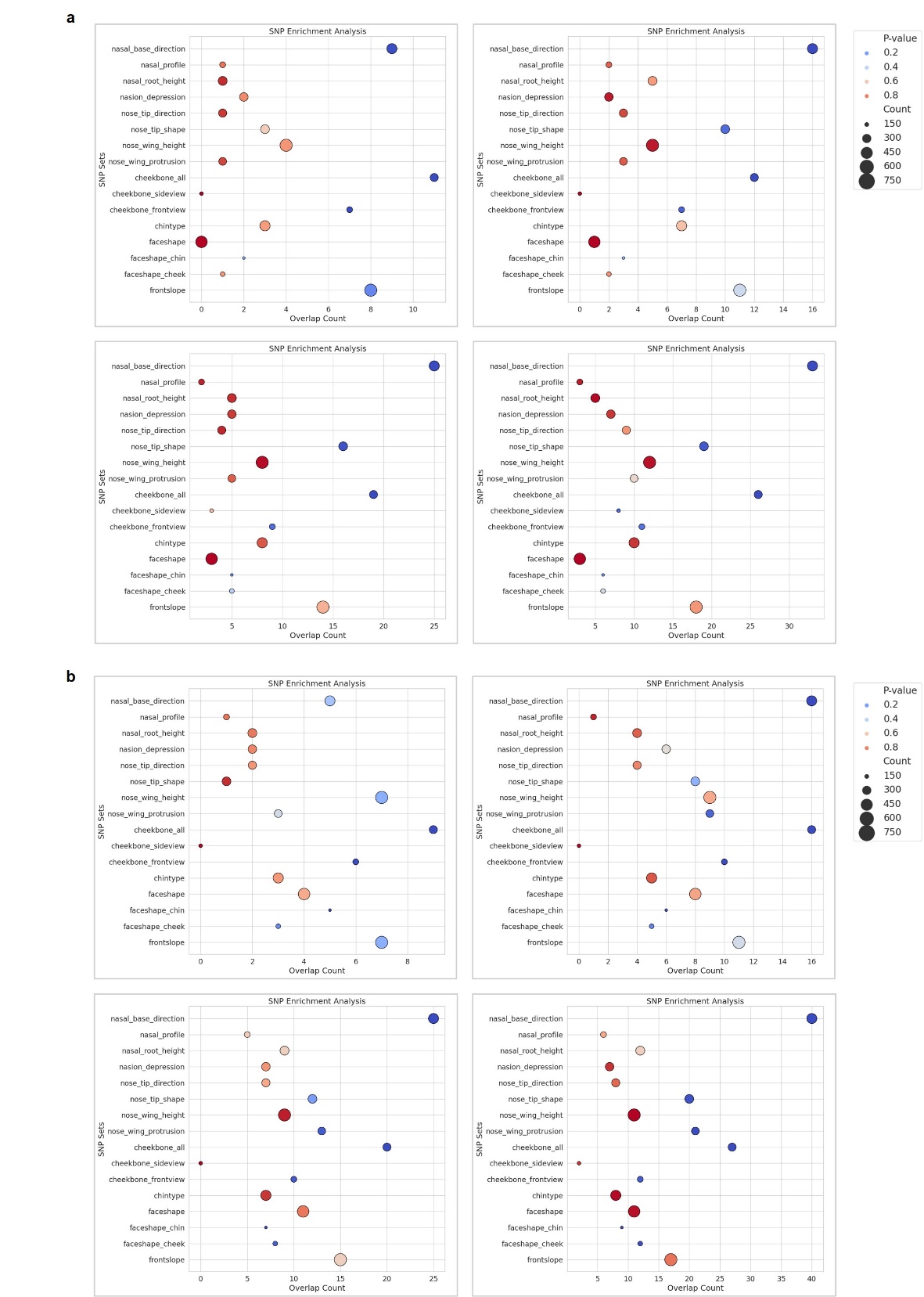


**Figure S5. SNP Enrichment Analysis in GWAS and SHAP Value Rankings.** This figure presents a series of SNP enrichment analyses comparing GWAS significance (p<0.05) with SHAP values. (a) Four panels depict the hypergeometric distribution of overlap between SNPs significant in GWAS and the top 50, 100, 150, and 200 positive SHAP values. (b) Similarly, four panels explore the relationship between GWAS significant SNPs and the top 50, 100, 150, and 200 SHAP values by absolute magnitude. Each plot is color-coded based on the p-value and the size of each dot represents the count of overlaps.

**
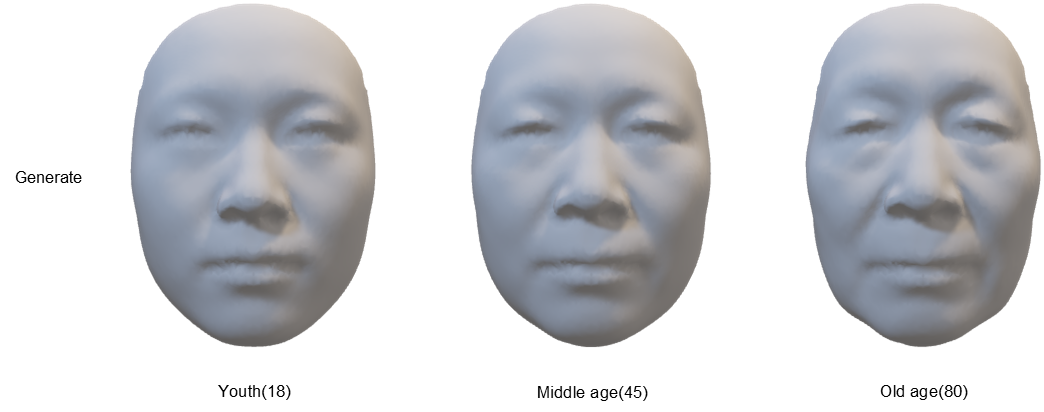
**

**Figure S6.** **Age-related 3D Facial Reconstructions: Progression from Youth to Old Age.** This figure illustrates the progression of facial morphology from youth to old age as modeled by the Difface system. The three faces represent individuals at three distinct age points: youth (18 years), middle age (45 years), and old age (80 years). Quantitative analysis of these transitions showed a facial change magnitude of 1.430 from youth to middle age, 1.689 from middle age to old age, and an overall change of 2.415 from youth to old age. These reconstructions highlight Difface's ability to simulate age-related transformations in 3D facial images.
